# Clinical and economic impact of multiple gated acquisition scan monitoring during anthracycline therapy

**DOI:** 10.1038/sj.bjc.6600037

**Published:** 2002-01-21

**Authors:** I Shureiqi, S B Cantor, S M Lippman, D E Brenner, M E Chernew, A M Fendrick

**Affiliations:** The University of Texas M. D. Anderson Cancer Center, Department of Clinical Cancer Prevention, 1515 Holcombe Boulevard, Box 236, Houston, TX 77030, USA; The University of Texas, M. D. Anderson Cancer Center, Department of Biostatistics, 1515 Holcombe Boulevard, Box 236, Houston, TX 77030, USA; The University of Michigan Health System and School of Public Health, Michigan, USA

**Keywords:** cost and cost analyses, multiple gated acquisition scan, anthracyclines, cardiotoxicity

## Abstract

The clinical and economic impacts of monitoring cardiac function in patients given doxorubicin have yet to be determined, especially in relation to patient age, cumulative doxorubicin dose, and the relative efficacies of doxorubicin-based *vs* alternative regimens. We developed a decision analysis model that includes these factors to estimate the incremental survival benefit and cost-effectiveness of using multiple gated acquisition scans to measure left-ventricular ejection fraction before and during doxorubicin chemotherapy. Probability distributions for the incidences of abnormal left-ventricular ejection fraction findings and congestive heart failure were derived from a retrospective review of 227 consecutive cases at The University of Michigan Medical Center and published findings. Multiple gated acquisition-scan monitoring minimally improved the probability of 5-year survival (<1.5% in the base–case scenario). For patients who received up to 350 mg m^−2^ of doxorubicin, multiple gated acquisition-scan screening had an incremental cost of $425 402 per life saved for patients between the ages of 15–39. This incremental cost markedly decreased to $138 191, for patients between the ages of 40–59, and to $86 829 for patients older than 60 years. The small gain in 5-year survival probability secondary to multiple gated acquisition scan monitoring doubled for all age groups when the average cumulative dose for doxorubicin reached 500 mg m^−2^. Variations in the cure rate differences between the doxorubicin and alternative regimens had insignificant effects on the improvement in 5-year survival rates from multiple gated acquisition-scan screening. The use of multiple gated acquisition scans for pretreatment screening appears to be more cost-effective for patients who are 40 years or older, when cumulative doxorubicin dose is 350 mg m^−2^ or less.

*British Journal of Cancer* (2002) **86**, 226–232. DOI: 10.1038/sj/bjc/6600037 www.bjcancer.com

© 2002 The Cancer Research Campaign

## 

Anthracyclines are common anticancer drugs that are active against a variety of human tumours, and tumour response to them is often dose dependent (Shan *et al*, 1996). Although effective, anthracyclines are also cardiotoxic and can induce acute, subacute, or delayed cardiac side effects (Steinherz and Yahalom, 1997). Irreversible subacute or early-onset chronic progressive cardiotoxicity (Giantris *et al*, 1998) are the most common forms of cardiotoxicity resulting from anthracycline administration and depend on both the cumulative dose and the administration rate (Von Hoff *et al*, 1979). Manifestation of these types of cardiotoxicity usually occur within a year of completing anthracycline therapy and produce mortality rates of up to 60% (Shan *et al*, 1996; Steinherz and Yahalom, 1997; Giantris *et al*, 1998). If the risk of anthracycline-induced cardiotoxicity could be accurately predicted, high-risk populations might be spared anthracycline exposure and low-risk patients might accrue maximum benefit from adequate doses of anthracyclines.

Monitoring the left ventricular ejection fraction (LVEF) with serial radionuclide ventriculography techniques such as multiple gated acquisition (MUGA) scanning can be used to estimate the risk of cardiotoxic reactions to anthracyclines (Stewart and Ratan, 1997). Schwartz *et al* (1987) reviewed clinical characteristics and LVEF (measured by serial resting radionuclide angiography) in 1487 cancer patients who were monitored with MUGA scanning during doxorubicin (Dox) chemotherapy. They identified patients at high risk for subacute cardiotoxicity and used their findings to propose guidelines for monitoring LVEF in patients undergoing Dox chemotherapy (Schwartz *et al*, 1987). Those guidelines involve a baseline LVEF measurement before the administration of 100 mg m^−2^ Dox. They also recommend subsequent measurements after cumulative doses have been given as follows: If the baseline LVEF is normal (>50%), the next LVEF measurement is conducted when the cumulative Dox dose reaches 250–300 mg m^−2^. Patients with a history of heart disease, radiation therapy, abnormal electrocardiographic results, or cyclophosphamide exposure should undergo a third LVEF measurement when the cumulative dose reaches 400 mg m^−2^; patients without these risk factors should undergo a third measurement when the cumulative dose reaches 450 mg m^−2^. LVEF measurement should be repeated before each subsequent dose (i.e., those that exceed 400 mg m^−2^ for the former group and 450 mg m^−2^ for the latter group). For all patients, if LVEF declines by 10% or more, Dox treatment should be stopped. If the baseline LVEF measurement is 30–50%, LVEF should be measured before each dose is given, and Dox should be discontinued if LVEF drops below 30%. If the baseline measurement is less than 30%, Dox should not be given at all.

These guidelines have several limitations. First, they do not account for the age of the patient, which is known to affect the risk of cardiotoxicities (Von Hoff *et al*, 1979). Second, the guidelines do not consider the contribution of Dox cumulative dose. The probability of Dox-induced cardiotoxicity has been shown to be minimal for cumulative doses of 350 mg m^−2^ or less, especially in patients less than 40 years old (Von Hoff *et al*, 1979). The total cumulative Dox dose in several common anthracycline-based regimens (e.g., Dox+cyclophosphamide (AC), Dox+bleomycin+ vinblastine+dacarbazine (ABVD), cyclophosphamide+Dox+vincristine+prednisone (CHOP)) is approximately 350 mg m^−2^, and the low probability of cardiotoxic reactions at this dose makes the benefit of pretreatment screening questionable for patients who are less than 40 years old. Third, the Schwartz guidelines were developed with a subset of 282 patients considered to be at high risk of developing congestive heart failure (CHF); most (62%) of these high-risk patients received Dox doses in excess of 450 mg m^−2^, whereas only 12% of the original group of 1487 were given doses that high. Thus, the benefit of MUGA-scan monitoring seen in the high-risk study cohort might not be applicable to patients who receive lower doses. In addition, the sensitivity and positive predictive value of MUGA-scan screening for Dox doses less than 100 mg m^−2^ in the Schwartz study were quite low (22% and 14%, respectively) (Schwartz *et al*, 1987). Finally, the proposed guidelines are demanding and compliance with them has been poor; according to one report, ‘many centres perform only a single LVEF measurement at an intermediate cumulative dose’ (Carrio *et al*, 1996).

An additional consideration that has yet to be addressed is the risk associated with using less-effective forms of therapy after a false-positive MUGA scan result, especially at the initial screening stage. Such a result might tend to favour the use of an alternative, non-anthracycline-containing regimen with inferior antitumour efficacy. Thus, the risk of reducing the rates of cure of cancer or response in the case of a false-positive MUGA scanning result should be considered.

We believe that guidelines for monitoring cardiotoxic reactions in patients given anthracycline chemotherapy should account for patient age, cumulative Dox dose, and the risk that a false-positive result may affect the choice of chemotherapy. To address these issues, we developed a decision analysis model which includes these factors and used that model to study the cost-effectiveness of monitoring LVEF with MUGA scanning during doxorubicin chemotherapy.

## METHODS

### Decision analysis model

We constructed a decision analysis model to assess the clinical benefit and cost-effectiveness of MUGA screening. All analyses were produced with Treeplan software (Decision Support Services, San Francisco, CA, USA). The base-case model was constructed for a patient considering chemotherapy for Hodgkin's lymphoma. The total Dox dose was assumed to be ⩽350 mg m^−2^, which is 50 mg m^−2^ above the average total dose for six cycles of ABVD chemotherapy. Three scenarios were analyzed to assess the effect of age: one for patients aged 15–39 years, one for patients aged 40–59 years, and one for patients aged 60 years or older.

**Figure 1:**
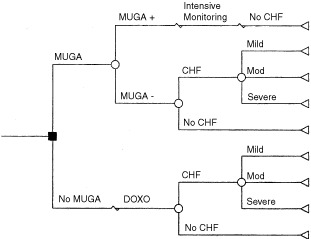
Decision analysis tree for evaluating the effects of MUGA scan monitoring on 5-year survival probability for patients who are treated with doxorubicin based chemotherapy.

The decision-analytic model (Figure 1

) begins with the decision whether to perform a baseline (screening) MUGA scan before chemotherapy. If a screening scan is performed, there are two possible outcomes: negative or positive scan results. If the screening MUGA scan is positive (i.e., the LVEF is less than 50%), intensive monitoring will occur with a repeated scan before each dose, as recommended by the guidelines. If the screening MUGA scan is negative and Dox is given, CHF may occur, in mild, moderate, or severe form. If a screening MUGA scan is not conducted, all patients are assigned to Dox therapy. Again, after Dox therapy, CHF may occur, with varying levels of severity.

### Data and assumptions

We reviewed the records of 227 consecutive patients who had MUGA-scan screening studies at The University of Michigan Medical Center between 1 July 1995 and 1 July 1996 to assess the likelihood of having a positive (LVEF <50%) MUGA scan before the start of Dox therapy.

For patients who underwent baseline MUGA-scan screening, we determined the likelihood of CHF occurrence and severity based on previously published studies. We assumed that the probability of CHF after a positive MUGA scan to be zero, given the findings of Schwartz *et al* (1987) that CHF was not found in any of the patients with abnormal baseline LVEF whose care followed Schwartz's guidelines. Schwartz *et al* (1987) also observed a sevenfold reduction in CHF incidence in patients whose care followed their proposed guidelines compared with patients whose care did not follow the guidelines. Therefore, for our model, we assumed that the probability of CHF occurring in a patient whose screening scan was negative was equal to the reported probability of CHF in patients given the same Dox dose but no LVEF screening measurement (Von Hoff *et al*, 1979), divided by seven. This calculation maximized the benefit of MUGA screening for this group.

For patients who did not undergo baseline MUGA-scan screening, we estimated the probabilities of CHF incidence and severity from a previous report of patients given 350 mg m^−2^ of Dox on a 3-week schedule who did not have LVEF measurements during treatment (Von Hoff *et al*, 1979). The probability of cardiotoxicity was adjusted by age using estimates from published data (Von Hoff *et al*, 1979).

### Clinical outcomes

We used 5-year survival as our clinical outcome measure. Several studies have shown that adding doxorubicin to the chemotherapy regimen for the treatment of Hodgkin's disease improves survival (Bonadonna *et al*, 1986; Radford *et al*, 1995). We incorporated published cancer survival rates for patients with Hodgkin's disease treated with chemotherapy that either included or did not include Dox from the study by Bonadonna *et al* (1986). According to these data, treatment with MOPP–ABVD, which includes Dox, produced a 76% 5-year disease-free survival rate, whereas treatment with MOPP, which does not include Dox, produced a 62% 5-year disease-free survival rate (Bonadonna *et al*, 1986).

To determine 5-year survival rates from CHF, we used data from a published meta-analysis (Dracup *et al*, 1994). We assumed that mild, moderate, and severe CHF (Von Hoff *et al*, 1979; Schwartz *et al*, 1987) corresponded to the New York Heart Association classes II, III, and IV, respectively, for use in our model. The 5-year mortality ranges associated with mild, moderate, and severe CHF are 25–50%, 50–100%, and 100%, respectively. Because the use of angiotensin-converting enzyme inhibitors has increased CHF survival rates (Kleber and Niemoller, 1993), we used the lower bounds of the mortality ranges to estimate CHF 5-year survival rates of 75%, 50%, and 0% (Dracup *et al*, 1994), (Table 1

**Table 1:**
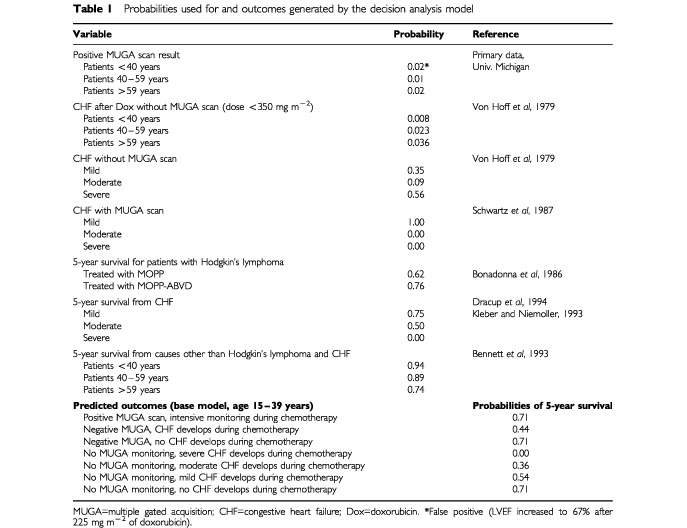
Probabilities used for and outcomes generated by the decision analysis model

).

Death rates from causes other than Hodgkin's disease and cardiotoxicity were derived from data published by Bennett *et al* (1993). In that study these death rates from causes other than Hodgkin's lymphoma or cardiovascular causes were reported as 6, 11, and 26% for patients aged <40, 40–59, and >60 respectively. The 5-year survival probability for all outcomes were multiplied by the survival probability from death by causes other than Hodgkin's lymphoma and CHF.

The survival rates for cancer and CHF are assumed to be independent, i.e., the probability of survival in each outcome category listed below is the product of the probability of cancer-specific survival multiplied by the probability of survival from CHF and the probability of death from other causes. If the decision is made to treat with a ‘no-Dox’ regimen, then the risk for developing treatment-related cardiotoxicity is assumed to be zero.

The outcome calculations were calculated as follows and the results are shown in Table 1: Patients with a positive (abnormal) screening LVEF result with Dox therapy and CHF: The probability distribution was considered to be zero, given the findings of Schwartz *et al* (1987) that CHF was not found in any of the patients with abnormal baseline LVEF whose care followed Schwartz's guidelines.

For patients with a positive (abnormal) screening result with Dox therapy without CHF: Survival was assumed to equal the probability of survival from cancer on a Dox-containing regimen multiplied by the survival probability from death by causes other than Hodgkin's lymphoma and CHF.

For patients with a negative (normal) screening result with Dox therapy with CHF: The probability of survival for each degree of CHF was estimated by multiplying the probability of survival from CHF by the probability of survival from cancer after ‘no-Dox’ chemotherapy (62%) and by the survival probability from death by causes other than Hodgkin's lymphoma and CHF. This group of patients has higher 5-year cancer relapse rates compared to a group receiving Dox therapy by approximately 27% (Bonadonna *et al*, 1986). This estimate was based on the assumption that baseline monitoring would predict the development of CHF early in treatment and prompts a rapid change to a ‘no-Dox’ chemotherapy regimen.

For patients with a negative (normal) LVEF result with Dox therapy without CHF: Survival was equal to the probability of survival from cancer after Dox-containing chemotherapy (76%) multiplied by the survival probability from death by causes other than Hodgkin's lymphoma and CHF.

Finally, for patients who were not scanned with Dox therapy with CHF: The probability of survival for each degree of CHF was estimated by multiplying the probability of survival from CHF by the probability of survival from cancer after Dox-containing chemotherapy and by the survival probability from death by causes other than Hodgkin's lymphoma and CHF. It was assumed that CHF detection would be delayed by the lack of monitoring and therefore a total Dox dose of 350 mg m^−2^ would be administered.

### Cost estimates

We calculated the economic costs associated with the clinical strategies of pretreatment MUGA-scan screening and no pretreatment screening. The cost of the MUGA scan itself was $751, the charge-list price at The University of Michigan Medical Center in 1996. Costs in terms of patient time were calculated by multiplying an estimated 3 h (for travel, waiting, and testing) by the average hourly earnings for each age group from a previously published population survey (Stinnett *et al*, 1996). The average hourly earnings were converted from 1993 dollars into 1996 dollars by using annual consumer price indices for health care expenditures. Annual costs for CHF-related care were based on a previously published cost-effectiveness analysis of pharmacological treatments for CHF: $8117 per patient per 10 years (Paul *et al*, 1994). Paul *et al* (1994) determined that the 10-year expected costs of CHF treatment using standard plus enalapril therapy is $8117 in 1992 dollars using a 5% discount rate. For our model, we needed to determine the expected costs of CHF treatment for a 5-year period in 1996 dollars using a 3% discount rate. A discount rate of 3% is currently the accepted discount rate, as given by a recent report of the U.S. Panel on Cost-Effectiveness in Health and Medicine (Gold *et al*, 1996). We ‘backed into’ an estimate of the annual cost of CHF therapy by assuming a constant annual survival rate over the 10-year period. We then converted the annual cost of CHF therapy in 1992 dollars into 1996 dollars by using annual consumer price indices for health care expenditures. Once again, we invoked the assumption of a constant annual survival rate and used a 3% discount rate to determine the total costs of CHF therapy for a 5-year period, as required by our model. The 5-year cost for CHF treatment was $6885. The costs of Dox-based and non-Dox-based chemotherapy were assumed to be equal except for patients who received ‘no-Dox’ chemotherapy regimen who have a higher relapse rate by an approximately 27% (Bonadonna *et al*, 1986). The cost for additional high dose salvage chemotherapy for the group was calculated by multiplying the number of excess cases requiring salvage chemotherapy by the cost of high dose salvage chemotherapy ($45 792) (Smith *et al*, 1997). Because only patients with good performance status are generally given chemotherapy, charges for caregiver time were not included in the cost calculation.

### Cost-effectiveness calculations

We calculated the incremental cost-effectiveness ratio (ICER) for MUGA-scan monitoring as compared to no monitoring as follows:


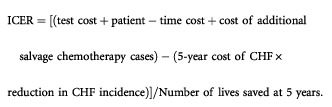


### Sensitivity analysis

Sensitivity analyses were used to assess the effects of cumulative dose and cure rates from Dox and non-Dox regimens on the outcomes of the model. Dox cumulative dose is an important predicator of the risk of cardiotoxicity and the risk increases markedly for doses of 500 mg m^−2^ and higher (Von Hoff *et al*, 1979). To assess the effect of higher cumulative doses on model outcome, we increased the average cumulative dose to 500 mg m^−2^ for the group that had no pretreatment screening but maintained the dose at an average of 350 mg m^−2^ for the monitored group. These choices were made on the basis of findings by Schwartz *et al* (1987) that the average cumulative dose for the group cared for according to the proposed guidelines was 348 mg m^−2^ and that for the patients whose care did not follow the guidelines the average cumulative dose was 512 mg m^−2^. This difference could have been due to a delay in detection of CHF in the second group that allowed them to receive a higher cumulative dose. To assess the effects of possible differences in cure rates between Dox and non-Dox regimens on model outcome, we varied in the model the overall cancer specific survival and the difference in cancer-specific survival between the Dox and non-Dox regimens. In one group of these analyses the overall cancer specific survival was assumed to be above 50% (typical rates for lymphomas), and the second group the cancer specific survival was assumed to be less than 50% (typical rates for leukaemia and sarcomas). We then varied the difference in cancer specific survival between the two regimens at 10–40% for overall cure rates below 50% and 10–70% for cure rates above 50%.

## RESULTS

### Frequency of positive screening MUGA scan by age groups

Of the 227 patients screened from The University of Michigan database, 47 patients (21%) were 15–39 years old; 98 (43%) were 40–59 years old; and 82 (36%) were 60 years or older. The screening MUGA scan was abnormal in only four cases (2%) – 2% (one out of 47 cases) in the youngest age group, 1% (one out of 98 cases) in the 40–59-year-old group, and 2% (two out of 82 cases) in the older than 60-year-old group. The one positive screening MUGA scan in patients who were less than 40 years old prompted a monitoring with serial MUGA scan (one scan after each dose). The positive screening scan is likely to have been a false-positive finding because the patient's LVEF increased to 67% after a cumulative Dox dose of 228 mg m^−2^.

### Cost-effectiveness by age

**Figure 2:**
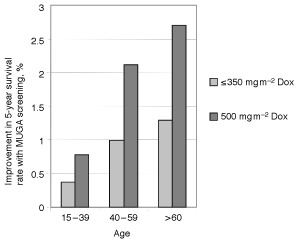
Improvement in 5-year survival rates with MUGA screening in patients given doxorubicin for two cumulative dose thresholds (⩽350 and 500 mg m^−2^).

Results from the base–case, in which patients were given an average Dox dose of 350 mg m^−2^, are shown in Figures 2

and 3

**Figure 3:**
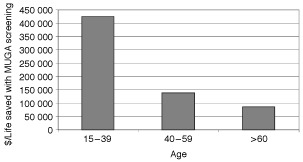
Cost-effectiveness estimates for MUGA scan monitoring in patients given doxorubicin dose of ⩽350 mg m^−2^ ($/life saved).

. Pretreatment MUGA scanning increased 5-year survival probabilities very modestly; the incremental improvement in survival rates were less than 1.5% for all three of the age groups. Patients younger than 40 years had the lowest improvement in their 5-year survival rates, approximately <0.4%, as shown in Figure 2. Cost-effectiveness was also dependent on age. Patients younger than 40 years old had the highest cost-effectiveness ratio ($425 402 per life saved, as shown in Figure 3). The incremental cost-effectiveness ratio dropped significantly for patients in the 40–59 age group ($138 191 per life saved), and for patients who were older than 60 years to $86 829 per life saved.

### Clinical outcome for MUGA scan monitoring and cumulative dose

The improvement in 5-year survival rates because of MUGA scan monitoring improved as the cumulative dose of Dox increased (Figure 2). For an average cumulative dose of 500 mg m^−2^, the small increments in 5-year survival probability because of MUGA-scan screening doubled for all age groups. The incremental improvement in 5-year survival probability reached 0.77% for patients between the ages of 15–39, 2.12% for patients between the ages of 40–59, and 2.7% for patients older than 60 years.

### Sensitivity analysis by cure rates

**Figure 4:**
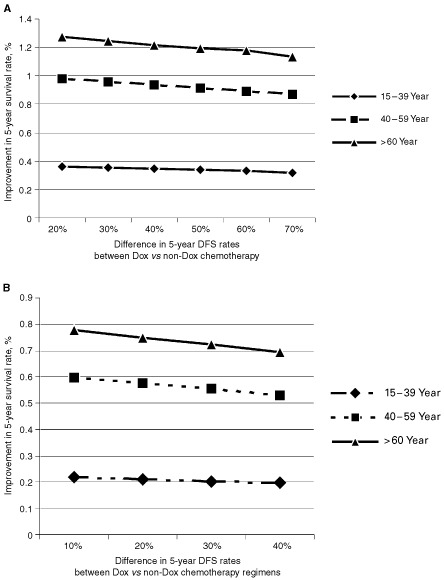
The benefit of MUGA scan monitoring in improving 5-year survival rates, with a cumulative doxorubicin (Dox) dose of ⩽350 mg m^−2^, in terms of different 5-year disease-free survival (DFS) rates for Dox *vs* non-Dox therapy. (**A**) A cost-effectiveness model was used in which the response rates for the Dox regimen was maintained at 76% and the response for the alternative non-Dox regimen was dropped in decrements of 10%. (**B**) A cost-effectiveness model was used in which the response rate for the Dox regimen was maintained at 46% and the response for the alternative regimen was dropped in decrements of 10%.

In contrast to age and cumulative dose, the differences in 5-year cure rates between Dox and an alternative chemotherapy regimen had very little effect on the clinical benefit from MUGA-scan monitoring (Figure 4A,B

). The overall cure rates from chemotherapy can vary significantly. For example, 5-year survival rates above 50% are common in Hodgkin's lymphoma (Bonadonna *et al*, 1986), while the expected cure rates for acute myeloid leukaemia are below 50% (Estey *et al*, 1997). We have examined two scenarios with our sensitivity analyses using a model for a cumulative Dox dose of <350 mg m^−2^. In the first case scenario the overall survival rate was fixed for the Dox regimen at 76% as in the base-case model while the rates for the alternative regimen were decreased by 10% for each subsequent analysis (Figure 4A). The 5-year survival benefit from MUGA scanning had minimal variation for all tested levels for all age groups (Figure 4A). In the second case scenario we assumed that the 5-year survival rate for the Dox regimen was 46% and we decreased the 5-year survival rates for the alternative regimen by 10% for each tested level. Again, there was minimal variation in the modest clinical benefit from MUGA scan monitoring for all age groups (Figure 4B).

## DISCUSSION

Our cost-effectiveness analysis confirmed that factors such as patient age and cumulative Dox dose influence the utility of MUGA-scan monitoring in patients given Dox chemotherapy. The decision analyses showed that age significantly affects the potential benefit of pretreatment MUGA-scan screening, with older patients benefiting the most. Our cohort included a significant proportion of young patients (21% were less than 40-years-old), as might be expected for a tertiary referral center; nevertheless, the single abnormal LVEF result in this subgroup turned out to be a false-positive finding. Even if we assumed that the false-positive scan was truly positive (as we did in this cost-effectiveness analysis), the overall improvement in 5-year survival probability was very small (less than 0.4%), and the cost-effectiveness ratio for MUGA scan monitoring for cumulative Dox dose of <350 mg m^−2^ for patients in this age range was much higher than in other age groups ($425 402 *vs* $138 191 *vs* $86 829 per life saved).

We recognize that assigning an economic value for survival improvement is difficult regardless of how small the differences in clinical benefit are. However, to put these results in perspective, we can compare these results to accepted measures to improve survival for patients with cancer. For example, patients with stage III colon cancer can expect their 5-year survival to improve by approximately 18% with adjuvant chemotherapy (Mamounas *et al*, 1999). Based on recently published data for an adjuvant chemotherapy cost analysis (Michel *et al*, 1999), the expected cost for adjuvant chemotherapy would be $5485 per patient and the expected cost per life saved at 5 years would be $30 472. We have found using our model that the cost per life saved at 5 years for patients who are younger than 40 years old and received Dox at a cumulative dose of ⩽350 mg m^−2^ is $425 402 which is 14 times higher than the cost for adjuvant chemotherapy for stage III colon cancer. In contrast, patients who were older than 60 years had a relative cost for MUGA scanning that is 2.85 times higher than the cost for adjuvant chemotherapy for stage III colon cancer.

Cumulative Dox dose was also a strong determinant of the clinical benefit of MUGA-scan screening, with most of the benefit associated with doses higher than 350 mg m^−2^. When the small survival benefit associated with monitoring was compared between two cumulative Dox doses, the survival benefit conferred by MUGA scanning was twice as high for the 500 mg m^−2^ group as for the 350 mg m^−2^ group. Furthermore, the effects of age and cumulative dose seemed to be additive, which further emphasized that patients who are younger than 40 and are given ⩽350 mg m^−2^ of Dox are the least likely to benefit from pretreatment MUGA scanning. All of these findings agree with those of Von Hoff *et al* (1979) on risk factors for Dox-induced CHF.

We observed very little impact for the difference in the 5-year survival rates between the Dox chemotherapy regimen and an alternative regimen on the clinical benefit from monitoring with MUGA scan. Our inability to detect these effects might have resulted from the very small overall benefit of MUGA scan. An additional factor one might also consider is that the model we used was biased in favour of maximizing the benefit of MUGA-scan monitoring through its assumption that Schwartz's findings with high-risk patients (Schwartz *et al*, 1987) could be generalized to all patients at all dose levels.

Our study has limitations that are typical of decision analysis studies, and our findings should be interpreted in light of these limitations. First, the probability distributions for the results of MUGA-scan screening were derived from retrospective data. Also, patients who are at risk for cardiotoxicity because of a history of coronary artery disease or CHF probably would not have been given Dox-based chemotherapy, and thus our findings may not be applicable to patients with pre-existing cardiac problems. We focused on subacute cardiotoxicity that develops within 12 months after the end of Dox therapy; the data used for the outcomes in the decision analysis models came from a study in which patients were followed for a mean of 14 months (Schwartz *et al*, 1987). Unfortunately, the cardiotoxic effects of anthracyclines can appear years or decades after Dox treatment ceases, and the sensitivity of MUGA-scan monitoring for detecting this type of delayed cardiotoxicity is very low (Shan *et al*, 1996). Thus, more sensitive screening methods are being sought.

Despite these limitations, we believe that our findings will be useful for improving the current guidelines for MUGA-scan screening. We believe that monitoring guidelines should account for patient age and cumulative anthracycline dose; our findings also highlight, for the first time, the overall small contribution of MUGA-scan monitoring to patient outcome, especially for Dox doses of 350 mg m^−2^ or less in young patients. We recognize that conducting prospective randomized studies in this area would be extremely difficult, and thus we used cost-effectiveness analyses to investigate the influence of these factors.

In summary, we demonstrated that the cost-effectiveness of using MUGA scans for monitoring anthracycline-induced cardiotoxicity depends on patient age and cumulative drug dose. Our findings suggest that the use of MUGA scanning for pretreatment screening of patients who are younger than 40 years, have no evidence of cardiac disease, and are given Dox at doses of 350 mg m^−2^ or less comes at a very high cost and has very minimal benefit for these patients. Future studies are needed to confirm these findings and to investigate the sensitivity, specificity, and positive and negative predictive value of MUGA scan monitoring for subacute and delayed anthracycline-induced cardiotoxicity.
